# Structural Characterization and Osseointegrative Properties of Pulsed Laser-Deposited Fluorinated Hydroxyapatite Films on Nano-Zirconia for Implant Applications

**DOI:** 10.3390/ijms23052416

**Published:** 2022-02-22

**Authors:** Min Li, Satoshi Komasa, Shigeki Hontsu, Yoshiya Hashimoto, Joji Okazaki

**Affiliations:** 1 Department of Removable Prosthodontics and Occlusion, Osaka Dental University, 8-1 Kuzuhahanazono-cho, Hirakata 573-1121, Japan; komasa-s@cc.osaka-dent.ac.jp (S.K.); joji@cc.osaka-dent.ac.jp (J.O.); 2 Department of Biomedical Engineering, Faculty of Biology-Oriented Science and Technology, Kindai University, 930 Nishimitani, Kinokawa 649-6493, Japan; hontsu@waka.kindai.ac.jp; 3 Department of Biomaterials, Osaka Dental University, 8-1 Kuzuhahanazono-cho, Hirakata 573-1121, Japan; yoshiya@cc.osaka-dent.ac.jp

**Keywords:** nano-zirconia, pulsed laser deposition, fluorinated hydroxyapatite, osseointegration, implant

## Abstract

Standard zirconia implants used in restoration still present problems related to inertness and long-term stability. Various physicochemical approaches have been used to modify the implant surfaces to improve early and late bone-to-implant integration; however, no ideal surface modification has been reported. This study used pulsed laser deposition to deposit a fluorinated hydroxyapatite (FHA) film on a zirconia implant to create a biologically active surface. The film prepared was uniform, dense, and crack-free, and exhibited granular surface droplets; it also presented excellent mechanical strength and favorable biological behavior. The FHA-coated implant was implanted on the femur of Sprague–Dawley rats, and various tests and analyses were performed. Results show that the in vitro initial cell activity on the FHA-coated samples was enhanced. In addition, higher alkaline phosphatase activity and cell mineralization were detected in cells cultured on the FHA-coated groups. Further, the newly formed bone volume of the FHA-coated group was higher than that of the bare micro-adjusted composite nano-zirconia (NANOZR) group. Therefore, the FHA film facilitated osseointegration and may improve the long-term survival rates of dental implants, and could become part of a new treatment technology for implant surfaces, promoting further optimization of NANOZR implant materials.

## 1. Introduction

Dental implants have become a priority and a reliable treatment option for patients with missing teeth [1,2], providing many advantages, including comfort, aesthetic enhancement, lack of damage to adjacent teeth, and significant clinical effects. With the developments in materials science, zirconia implants have been employed to overcome problems with metal allergies and the gray color of titanium implants [3,4,5], providing patients with a new choice for dental implant materials [6,7]. In the process of implant repair, mechanical properties [8] and osseointegration [9] are key factors that ensure the success and long-term stability of the implant. Studies show that when zirconia implants are tested for static and cyclic fatigue under laboratory conditions, they can withstand the shear force required for implantation and show better mechanical properties than titanium implants [10,11,12]. In a recent study, the mechanical properties of zirconia were improved by adjusting its microstructure [13].

In this study, micro-adjusted composite nano-zirconia, referred to as NANOZR [14,15], was chosen. It has an excellent aesthetic effect, a good aging stability for resisting low-temperature addition degradation (LTAD), and the best mechanical properties because of its nanostructure. However, the problem of surface inertia must be addressed to improve the osseointegration. Zirconia has been lauded as an inert biomaterial [16], although the adsorption of blood protein and platelets, and the migration of cells, suggest a biological interaction with zirconia-based surfaces [17]. 

Current surface modification technologies used for zirconia implant fabrication mainly include coating and physicochemical methods [18,19,20,21,22,23,24,25,26]. Physicochemical methods include sand blasting, acid etching, ultraviolet irradiation, laser surface modification, and plasma treatment, although there may be a risk of reducing the mechanical properties of zirconia implants with these approaches. The coating method includes the use of bioactive glass, polydopamine (PDA) coating, and apatite coating, which presents a risk of stripping and degradation. The limitations of the existing technology creates the need to develop a new surface-treatment technology.

Previous studies have successfully prepared a hydroxyapatite film on the material surface by pulsed laser deposition (PLD) [27]. This technology may be used to prepare films with consistent chemical composition, high continuity, compact structure, and high bonding strength between the film and matrix.

Taking advantage of the superiority of this technology and based on the fact that hydroxyapatite (HAp) [28,29,30] presents good biocompatibility, bone induction ability, and low solubility of a fluorinated hydroxyapatite (FHA) film, a fluorinated hydroxyapatite film was prepared on the NANOZR surface to transform the implant surface into a biologically active surface, to see if the film would exhibit a certain bonding strength with the implant. In a previous study [31], a thin FHA film was successfully fabricated and optimum annealing conditions were identified; the same film-depositing conditions were used in this study, while microstructure and element analysis were performed to confirm whether the film was successfully deposited. To evaluate the implant surface bioactivity and osseointegration after the FHA film coating modification, in vitro and in vivo investigations were conducted. The purpose of the present study is to combine bioactive coatings and the NANOZR material to enhance its biological behavior and facilitate early osseointegration, to achieve long-term bone-to-implant contact and improve dental implant survival rates.

## 2. Results

### 2.1. Microstructural and Mechanical Properties of Materials

#### 2.1.1. Surface Morphology of NANOZR and FHA Coatings

**Figure 1:**
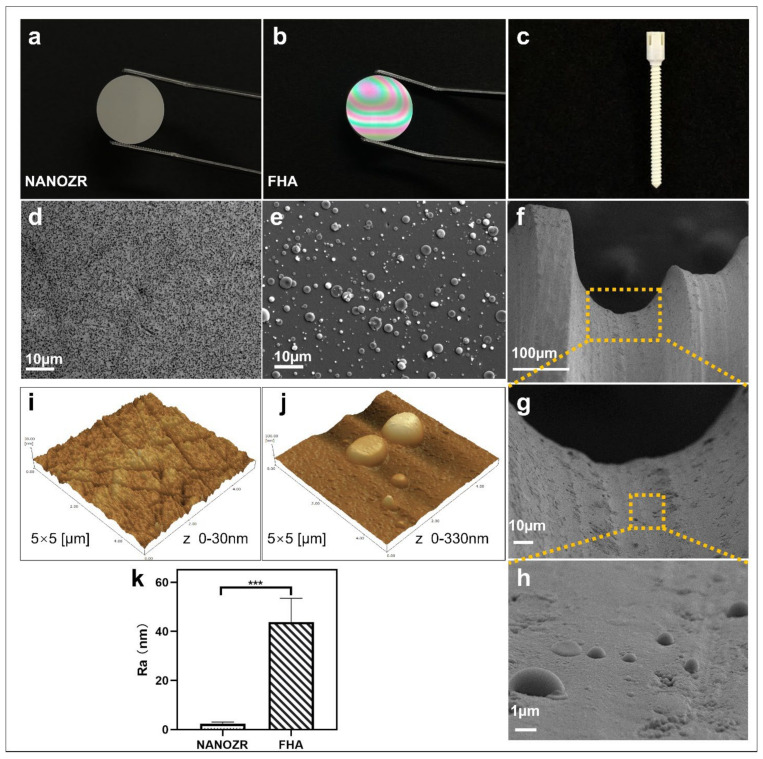
(**a**–**c**) Gross appearance of the NANOZR and fluoridated hydroxyapatite (FHA)-coated NANOZR Materials; (**d**,**e**) Scanning electron micrographs of the NANOZR and FHA-coated NANOZR disks; (**f**–**h**) Scanning electron micrographs of the FHA-coated screws; Partial magnifications of the orange rectangular area are displayed in the lower panel. (**i**–**k)** Scanning probe micrographs of the NANOZR and FHA-coated NANOZR surfaces. Roughness values (Ra) of the NANOZR and FHA-coated NANOZR disks, *** *p* < 0.001.

The gross appearance of uncoated NANOZR and FHA-coated materials is shown in Figure 1a–c. The microscopic structure of the NANOZR and FHA-coated disks is shown in Figure 1d,e, in which the black dots depict Al_2_O_3_ particles, and the white dots are zirconia particles. The FHA coating is uniform, without cracks, and exhibits a granular surface with the presence of droplets (2–3 μm). With respect to the droplets, pulsed laser interaction with the target causes a local heating and a fast emission of vapor, clusters, micro-grains, or particulates. For this reason, the occurrence of particulates on the surface is an inherent artifact of the PLD technique. The microstructure of the FHA-coated implant showed the same droplets (Figure 1f–h), and is characterized by a dense, homogeneous, and non-porous film. Figure 1j,k shows an Ra of 2 nm on the NANOZR disk surfaces, and the Ra of the FHA coating increases on average to 44 nm, which was significantly higher due to the presence of the FHA films with droplets. A similar surface morphology was previously observed on the surface of an HA film that was deposited by KrF laser ablation [19].

#### 2.1.2. Composition Analysis by EDX and XPS

**Figure 2:**
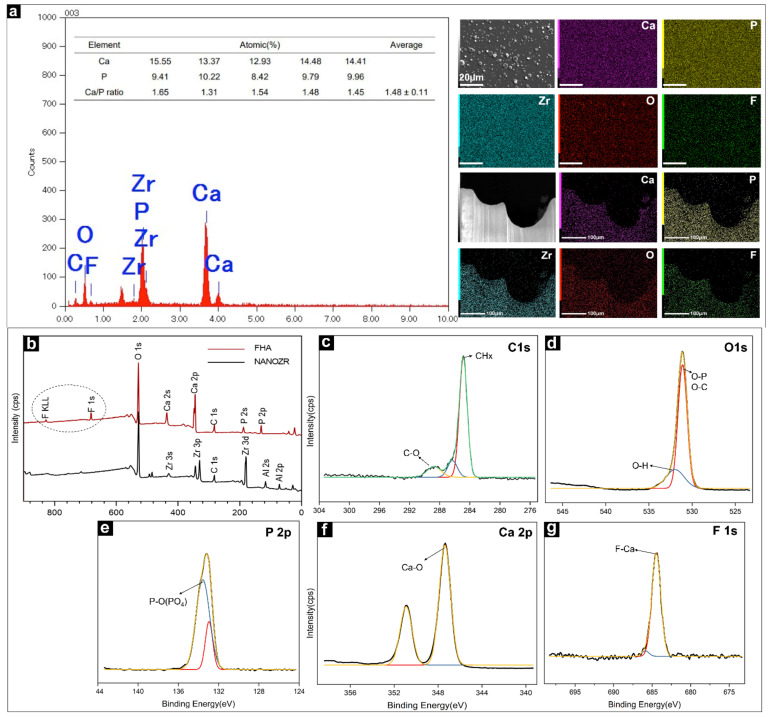
(**a**) EDS image of the FHA-coated NANOZR disks for elemental composition and weight percentage diagram; Elemental mapping (Ca, P, Zr, O, F,) of the FHA-coated NANOZR materials. (**b**) Wide scan of the XPS spectrum of NANOZR and FHA-coated NANOZR disks. High-resolution spectra for (**c**) C 1 s, (**d**) O 1 s, (**e**) P 2p, (**f**) Ca 2p, and (**g**) F 1s.

The EDX method was used to identify the distribution of elements present in the FHA films, as shown in Figure 2a, and it confirmed the uptake of different ion species. Ca, P, O, C, and F were identified as the main components of the FHA coatings. Zirconium was not detected, confirming that a complete film was deposited on the surface of the NANOZR substrate. The Ca/P ratio of FHA (1.48) was lower than that of stoichiometric HA (1.67). The results of the element mapping of the FHA-coated disks and implant are shown in Figure 2a. Ca, P, C, O, F, and Zr species are evenly distributed, which shows that the FHA coating deposited on the implant surface is uniform and completely covered. 

The wide-spectrum of the FHA coating (as produced at room temperature (20–25 °C)) are presented in Figure 2b. Zr3d elements were not found in the FHA coating. The results of the XPS analysis also confirmed that the FHA coatings were successfully deposited on NANOZR, and that the FHA coating completely covered the surface of the material. The data were collected using a high-resolution mode in the energy regions of C 1 s, O 1 s, P 2p, Ca 2p, and F1s (Figure 2c–g). The binding energies (BE) corresponding to Ca 2p, P 2p, and O 1s were 347.3 eV, 133.2 eV, and 531.1 eV, respectively. The F1s peak at 684.4 eV was also found, which is typical of F1s in the FHA structure, and the C1s peak at 284.6 eV and 289.1eV, was attributed to organic surface contamination (CHx) and inorganic (CO_3_^2−^), respectively.

#### 2.1.3. Identification of Functional Groups by FTIR

**Figure 3:**
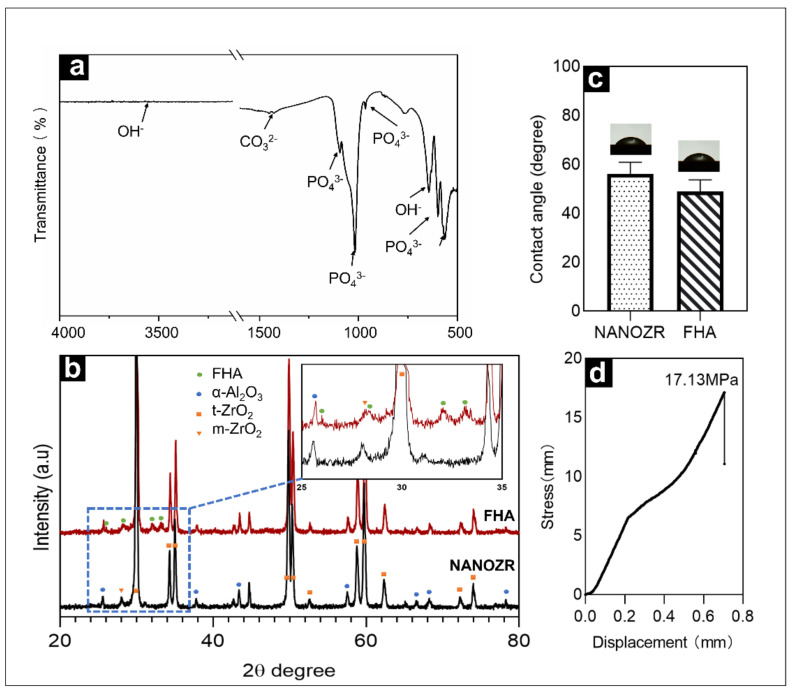
(**a**) Fourier transformed infrared spectra of FHA-coated NANOZR disks; (**b**) XRD patterns of NANOZR and FHA-coated NANOZR disks heat-treated at 450 °C for 10 h; (**c**) Contact angle of NANOZR and FHA-coated NANOZR disks; (**d**) Stress-displacement curves of the FHA-coated NANOZR disks.

Figure 3a shows the infrared spectrum of the FHA film within the wavenumber range from 500–4000 cm^−1^. The material exhibited characteristic peaks of PO_4_^3−^ at 1093 cm^−1^, 1018 cm^−1^, 964 cm^−1^, 599 cm^−1^, and 563 cm^−1^, exhibited characteristic peaks of OH^−^ at 3540 cm^−1^, and exhibited characteristic peaks of CO_3_^2−^ at 1452 cm^−1^, 1427 cm^−1^, and 875 cm^−1^. The reason for the appearance of the CO_3_^2−^ peak is that during sintering, CO_2_ in the air inevitably reacted with the target to form CO_3_^2−.^

#### 2.1.4. Crystalline Phase Identification by XRD

Figure 3b exhibits the XRD patterns of FHA films deposited on ΝAΝOΖR after heat treatment at 450 °C for 10 h. The sample were comprised of the α-Al_2_O_3_, t-ZrO_2_, few m-ZrO_2_ crystal phase diffraction peaks and FHA crystal phase diffraction peaks.

#### 2.1.5. Surface Wettability Determined by Contact Angle

The results shown in Figure 3c indicate that the hydrophilicity of the surface is improved after the film is deposited on the surface, with a decrease in the contact angle from 55.98° to 48.83°.

#### 2.1.6. Mechanical Properties Determined by Bond Strength Testing

The load rate used in these tests was 5 kg/s, with a stress rate of approximately 17.13 MPa (Figure 3d). 

### 2.2. Bioactivity Evaluation of NANOZR-FHA Coatings

#### 2.2.1. Cell Morphology and Viability

**Figure 4:**
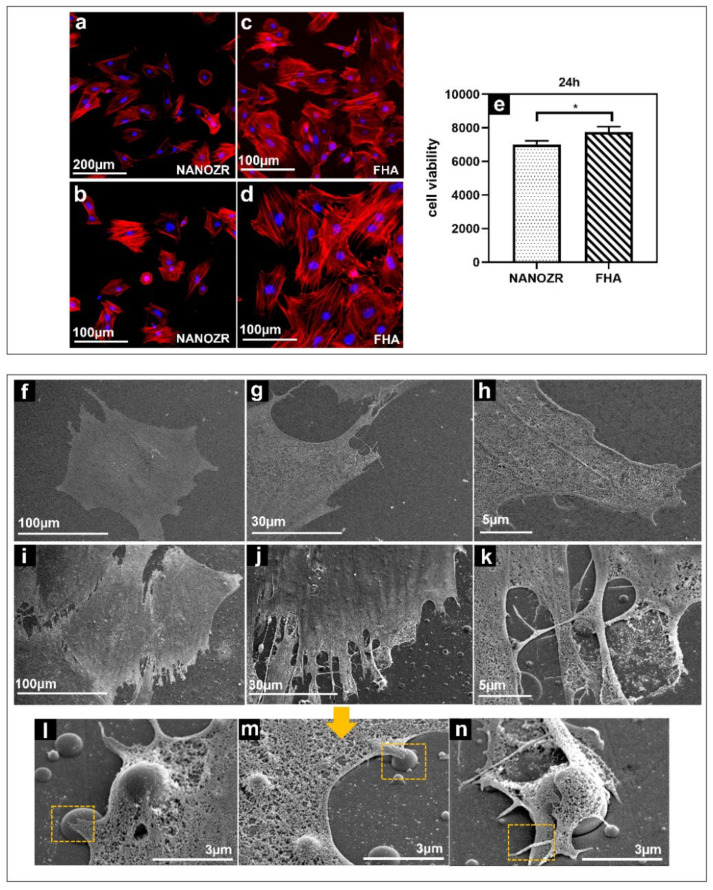
Morphological analysis of rBMMSCs attached to NANOZR (**a**,**b**) and FHA-coated NANOZR (**c**,**d**) disks after culturing for 24 h. Actin filaments (red) were labeled with Alex Fluor 594-phalloidin and nuclei (blue) were stained with 4′,6-diamidino-2-phenylindole; Cell viability of rBMMSCs attached to NANOZR and FHA-coated NANOZR (**e**) disks after culturing for 24 h, * p < 0.05. SEM analysis of the morphology of rBMMSCs attached to NANOZR (**f**–**h**), and FHA-coated NANOZR (**i**–**n**) disks after culturing for 24 h; Pictures at lower magnification (**i**–**k**) show the morphology of single cells.; Pictures at higher magnification (**l**–**n**) show the detailed interaction the cell with the FHA coating. (marked with the orange rectangular area).

The morphology of the rat bone marrow mesenchymal stem cells (rBMMSCs) on the NANOZR surface and the FHA coatings after 24 h of culturing, were observed using a fluorescence microscope, shown in Figure 4a–d). The cells were confirmed to adhere to the material surface in all groups. An increase in the number of cells and an elongation in cell projections was observed on the material surface of the FHA-coated materials. SEM observations are shown in Figure 4f–n, which indicate that several filamentous pseudopodia extended and closely adhered to the surface of the FHA-coated materials. These results imply that FHA coatings can promote cell growth and shows good biological activity (Figure 4e).

#### 2.2.2. Evaluation of Hard Tissue Differentiation

**Figure 5:**
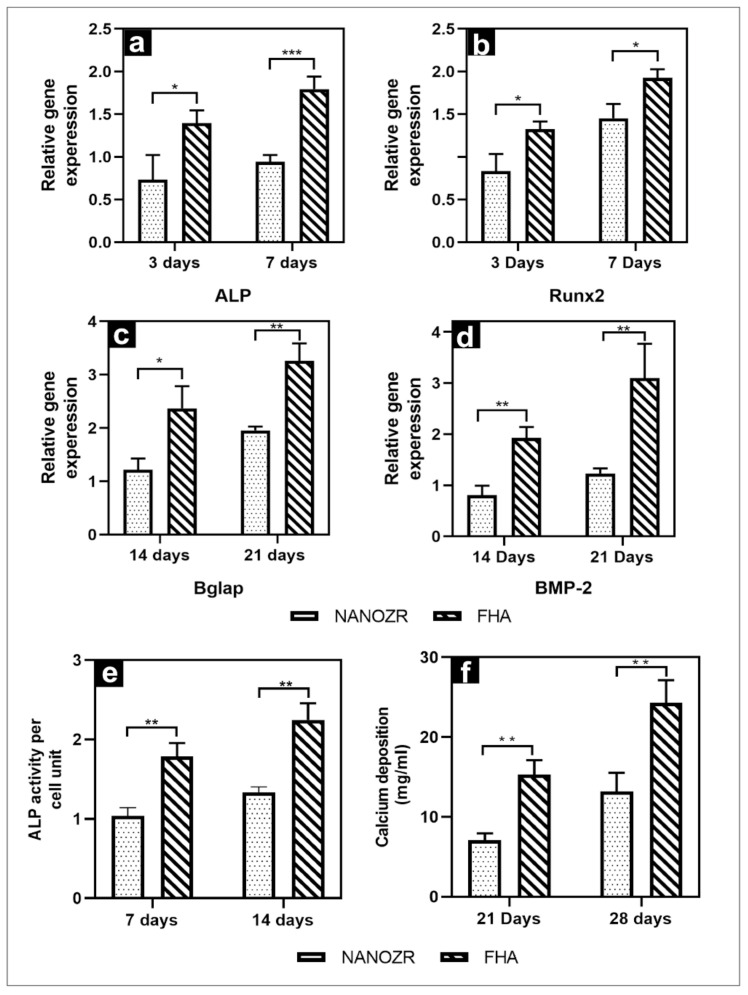
(**a**–**d**) Quantitative real-time (qRT)-PCR analysis of osteogenesis-related gene expression in NANOZR and FHA-coated NANOZR; (**e**) Alkaline phosphatase activity in NANOZR and FHA-coated NANOZR; (**f**) Calcium deposition in NANOZR and FHA-coated NANOZR, * *p* < 0.05, ** *p* < 0.01, *** *p* < 0.001.

The gene expression related to the induction of hard tissue differentiation on the material surface of the FHA-coated and NANOZR disks was analyzed. The mRNA expression levels of osteogenesis-related genes including alkaline phosphatase (ALP), runt-related transcription factor (Runx2), bone morphogenetic protein 2 (BMP-2), and osteopontin (OPN) in cells grown on the different surfaces for 3, 7, 14, and 21 d were assessed by qRT-PCR. A significantly higher gene expression was observed on the material surface of the FHA-coated disks at all measurement times (Figure 5a–d). 

The quantitative results of ALP activity are shown in Figure 5e. The ALP expression was measured as the initial marker for the induction of hard tissue differentiation. The relative ALP activity of the FHA-coated disks was significantly higher than that of the NANOZR disks on Days 7 and 14. Mineralization was assayed for calcification, which is a late marker for the induction of hard tissue differentiation. The amount of Ca deposited at 21 and 28 d after the incubation was significantly higher on the material surface of the FHA-coated disks (Figure 5f).

#### 2.2.3. Quantification of New Bone Formation

**Figure 6:**
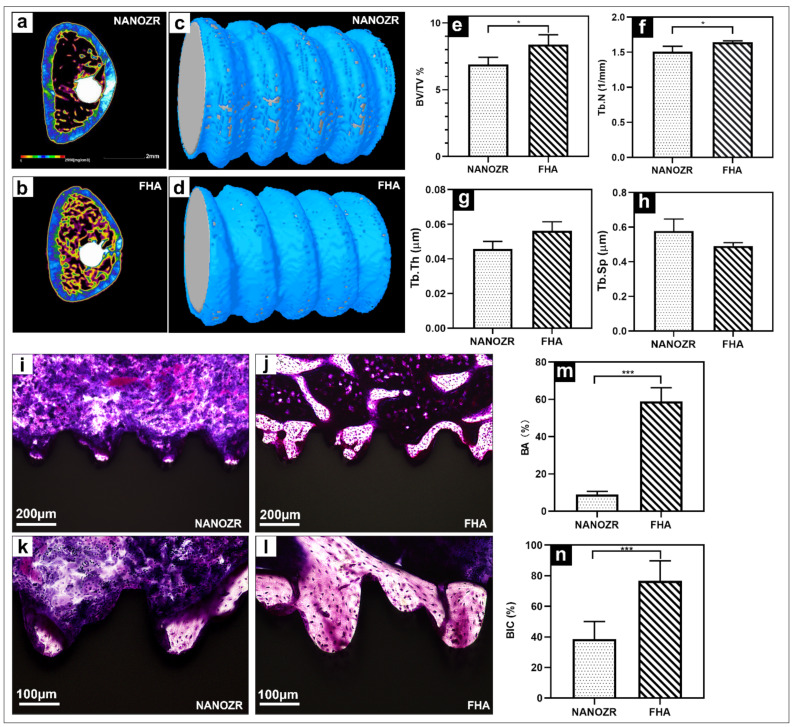
(**a**,**b**) Transverse reconstructed microcomputed tomography of the NANOZR and FHA-coated NANOZR implants after 8 weeks; (**c**,**d**) Reconstructed three-dimensional micro-CT images of bone tissues around the NANOZR and FHA-coated implants; (**e**–**h**) Bone volume to total volume ratio (BV/TV), trabecular number (Tb.N), trabecular separation (Tb.Sp), and trabecular thickness (Tb.Th) around the NANOZR and FHA-coated NANOZR implants after 8 weeks. (**i–l**) Histological sections of bone tissues around the NANOZR and FHA-coated implants; (**m**,**n**) Bone area ratio (BA) and bone implant contact (BIC) around the NANOZR and FHA-coated implants, * *p* < 0.05, *** *p* < 0.001.

The three-dimensional reconstructed microcomputed tomographs of femurs with implants are shown in Figure 6a,b, with the cortical bone shown in green, the cancellous bone in yellow, and the implant in white. 

The reconstructed three-dimensional micro-CT images showed a dense new bone layer formed on the FHA-coated implant (Figure 6d). The ratio of the bone volume to the total volume (BV/TV), mean trabecular number (Tb.N), and mean trabecular thickness (Tb.Th) were significantly higher in the FHA-coated implant, suggesting accelerated osteogenesis in the region of interest (Figure 6e–h, * *p* < 0.05). In contrast, the mean trabecular separation (Tb.Sp) was lower at both time points.

The histological sections are shown in Figure 6i–l, there was no gap between the bone tissue and the implant, and the number of new bone cells formed more on the FHA-coated implant. Quantitatively, the bone area ratio (BA) and bone implant contact (BIC) were significantly higher in the FHA-coated implant in Figure 6m,n.

## 3. Discussion

In this study, a new surface-treatment technology was described for zirconia implants that was developed to ensure good biological activity and osteoinductive ability to achieve osseointegration. The results suggest that the proposed approach could become a suitable implant surface modification technology and could be applied in a clinical setting in the future.

In recent years, zirconia implants have been gaining increased attention because of their excellent mechanical properties, bioactivities, and aesthetics, and many surface modification treatments have been performed on zirconia implants to achieve osseointegration [20]. Although modification will improve its osteogenesis ability, it weakens the mechanical properties, or long-term osteogenesis cannot be guaranteed. In other words, although surface modification can improve osseointegration, it does not have stability and long-term benefits [32,33,34]. The FHA film has proved to be a good bioactive coating [35,36], but the stability and uniformity of the film, the complexity of the coating approaches, and the practicability are limitations [37,38].

This study used PLD, which can overcome the aforementioned shortcomings to fabricate a 1 μm film (from previous experiments, the thickness of the film deposited at 6000 shots was approximately 1 μm [39]). The tensile test results showed that the adhesive strength of the film was 17 MPa. A previous report showed that the bonding strength between the HAp-coated titanium and the bone tissues is approximately 3.5 MPa [40]. The SEM, XPS, FTIR, and XRD results obtained in this study demonstrate that the FHA film was successfully deposited on NANOZR. However, the characteristic diffraction peak of the zirconia substrate was too high, and the film was thin, whereas the characteristic peak of FHA was not clearly visible in the spectrum. During the cooling period of the sintering schedule, the thermal expansion mismatch of NANOZR and the FHA film sometimes caused residual stress that exceeded a critical limit and resulted in cracks and delamination [41,42,43], subsequently influencing the growth of FHA. Further experiments are required to find the optimal sintering temperature.

After the FHA film coating, the roughness of NANOZR increased from 2 to 43 nm. Previous studies have documented that roughness has a strong influence on cell attachment [44]. Moreover, an increase in roughness can ensure favorable conditions for the promotion of osteoblast functions, leading to the formation of new bone [45]. The mapping and EDX results showed the presence of F in the FHA film, and a low dose of fluoride was reported to stimulate bone formation and increase bone mass without causing any mineralization defects [46,47]. The incorporation of fluoride into biological apatite may cause diverse changes, ranging from crystallographic alterations to an impact on biological performance. F^−^ can replace OH^−^, improve the crystallinity of the crystal, and improve the acid resistance of the film.

The results of the contact angle experiments suggest that the angle of the FHA-coated materials was reduced to 48°, and the hydrophilic performance was improved. Observing the morphology of the cell collagen skeleton by fluorescence microscope and electron microscope, the FHA-coated materials have a larger extension area and more antennae than the control group. Under high magnification, it can be observed that the tentacles of the cells in the experimental group tightly wrap around the spherical particles of the film, which is consistent with the conclusion that the surface roughness of the particles facilitates cell attachment. The results of cell viability experiments also showed that the cell viability of the FHA-coated materials was enhanced. Overall, the FHA films exhibited good mechanical properties and increased cell proliferation. Indeed, surface properties, roughness, and composition are major determinants of the cellular response to implants [48,49].

In addition, in the human body, the film dissolution phenomenon will make it controversial, releasing a variety of ions. Studies have shown that fluoride ions can not only prevent caries, but also promote the mineralization and crystallization of calcium phosphate during bone formation [50,51], and calcium ions can accelerate the calcification, deposition, and the formation of calcium nodes [31], which are consistent with our results. Furthermore, the real-time PCR showed that the expression of the Runx2, ALP, BMP-2, and Bglap genes in the FHA-coated materials was extremely high. In early osteogenesis, the transcription factors, ALP and Runx2, were abundantly expressed [52,53], meanwhile BMP-2 was expressed in the advanced stages [54]. These results imply that the FHA film has a significant enhancing effect on osteogenesis and biocompatibility while retaining the properties of HA.

In the in vivo experiments, the FHA-coated screws implanted into rat femurs exhibited a denser new bone, and the ossified areas in the sagittal view were significantly larger. These results showed that the quantity of newly formed bone in contact with the implants of the FHA-coated materials was drastically higher than that of untreated NANOZR. These results indicate that the FHA-film-coated materials promote the growth of osteoblasts and gives rise to in vitro bone formation, osteoblast proliferation, and differentiation [36]

At present, surface modification is used to increase roughness, which promotes osteogenesis, but the rough surface is also prone to bacterial adhesion to form biofilms and cause planting failure [55]. However, there is evidence suggesting that the FHA film can also have antibacterial properties [56]. Moreover, the antibacterial activity of FHA coatings is much higher than that of the pure hydroxyapatite (HA) coating and acid-etched pure titanium surfaces, and the coatings are suitable for orthopedic and dental applications.

Although important discoveries have been revealed by these studies, there are also limitations to the proposed approach. Regarding PLD technology, it is still necessary to solve the problem of mass production. In summary, it was identified that the FHA film prepared by the proposed technology can currently be used as a feasible option for the surface modification of zirconia implants while achieving the optimization of NANOZR implants. It has also been confirmed that this is an ideal surface-treatment technology for oral implants. In the future, we will simulate the complex environment in the body to explore other characteristics of FHA films and provide more evidence for clinical applications.

## 4. Material and Methods

### 4.1. Material Fabrication

NANOZR discs (diameter, 15 mm and thickness, 1.5 mm; Yamamoto Kinzoku, Osaka, Japan) and screw implants (external diameter, 1.2 mm and length, 12 mm) were used. These materials were cleaned ultrasonically with acetone, rectified spirit, and distilled water, and subsequently dried prior to the FHA (fluorinated hydroxyapatite)-coating treatment. Disk-shaped targets (diameter, 16 mm and thickness, 3 mm) were fabricated for PLD by pressing FAP (Fluoride apatite) powders (Taihei Chemical Industrial) at 20 MPa and sintering at 750 °C for 10 h. The deposition of fluorapatite onto the NANOZR discs and screw implants was performed by a krypton fluoride excimer laser (COMPexPro 205; wavelength [λ] = 248 nm and pulse width [τ] = 20 ns) operating at a repetition rate of 10 Hz in an atmosphere at 1 × 10^–5^ Pa. The substrate was at room temperature (20–25 °C), and the deposition rate was 10 nm/min. The film thickness was estimated based on the deposition rate; the thicknesses of the prepared FHA films were approximately 1 μm. These materials were then annealed by heating at 450 °C for 10 h, at heating and cooling rates of 1.5 °C/min. Before the analysis and in vitro experiments, the NANOZR and FHA coatings were cleaned using a series of 10 min ultrasound treatments in acetone, rectified spirit, and distilled water. For in vitro and in vivo experiments, all samples were sterilized in an autoclave at 160 °C for 3 h.

### 4.2. Surface Characterization

The surface morphology and microstructure of FHA coatings were observed mainly by secondary electron imaging using a scanning electron microscope (SEM, JSM-6510, JEOL, Tokyo, Japan) equipped with an energy-dispersive X-ray spectroscope (EDX) with an accelerating voltage of 15 kV. For the SEM observations, the samples were sputter-coated with an extremely thin, electrically grounded layer of an Au–Pd metallic alloy. 

Atomic force microscopy (AFM, SPM-9600; Shimadzu, Tokyo, Japan) was performed to obtain the mean average surface roughness (Ra) and three-dimensional surface topography of the FHA coatings. 

The surface chemical states and elemental composition were determined by X-ray photoelectron spectrometry (XPS; PHI X-tool; ULVAC-PHI, Kanagawa, Japan).

X-ray diffraction (XRD, Ultima IV, Rigaku, Japan) analysis was conducted with a Rigaku rotating anode diffractometer using Cu Kα radiation at 40 kV and 100 mA to check the crystallinity of the deposited and annealed coatings. Spectra were recorded within the range from 2θ = 3°–80°, a scanning speed of 2°/min and incident angle of 1°.

Fourier transform infrared spectroscopy (FTIR, IRAffinity-1S; Shimadzu, Kyoto, Japan) was performed to confirm the presence of the –OH, –CO_3_^2−^, and –PO_4_^3−^ functional groups.

The water contact angles on the samples were measured using a contact angle measurement system (VSA2500 XE; AST Products, Billerica, MA, USA) by the application of 2 μL of ddH2O to the surface.

The coatings were tested to determine their bond strengths using a *Z*-axis pull test. A stainless-steel rod with a diameter of 3 mm was glued onto the FHA film using epoxy glue. A stainless-steel rod was attached to the jig of a tensile tester (EZ-test, Shimadzu, Kyoto, Japan.) using a universal joint. A tensile load was applied to the specimen at a tensile rate of 0.5 mm/min until failure occurred.

### 4.3. Cell Culture

Experiments were performed in compliance with the National Animal Care Guidelines (approval no. 20–08004, 31 July 2020). Femurs were isolated from two male rats aged eight weeks, clipped at both ends, and flushed with media using a 21-gauge needle to collect the bone marrow. Bone marrow mesenchymal stem cells were then cultured in 75 cm^2^ flasks according to a well-documented method [31]. Primary cells were cultured at 37 °C in a 5% CO_2_ atmosphere in a growth medium containing minimal essential medium (Nacalai Tesque, Inc., Tokyo, Japan) supplemented with 10% fetal bovine serum (Nacalai Tesque, Inc.) and antibiotic–antimycotic mixed stock solution (Nacalai Tesque, Inc.). The media were changed every three days. rBMMSCs were removed from the flasks at the 3rd–5th culture and seeded at a cell density of 4 × 10^4^ cells/well onto the specimen surface.

### 4.4. Cell Morphology and Viability

After culturing for 24 h, samples were washed with phosphate buffer saline (PBS), fixed by incubation in 4% paraformaldehyde solution for 20 min at room temperature (20–25 °C), and permeabilized with 0.2% (*v*/*v*) Triton X-100 for 30 min at room temperature (20–25 °C). The cells were then incubated with the blocking one reagent (Nacalai Tesque, Kyoto, Japan) for 30 min at room temperature (20–25 °C) and stained with Alexa Fluor 594-phalloidin and 4′, 6-diamidino-2-phenylindole (DAPI) at 37 °C in darkness for 1 h. F-actin and cell nuclei were examined using a confocal laser scanning microscope (LSM700; Carl Zeiss, Oberkochen, Germany).

For the SEM analyses, rBMMSCs cells (1 × 10^5^ cells/well) were seeded onto the prepared NANOZR and FHA samples in a 24-well plate. After culturing for 1 d, the cells were washed three times with PBS, pH 7.4 (Gibco™, Thermo Fisher Life Technologies Ltd., Tokyo, Japan), fixed with 2% glutaraldehyde for 2 h, and then dehydrated using a series of ethanol concentrations (50, 60, 70, 80, 90, 99, and 100%). The samples were dried in a critical point dryer (HCP-1; Hitachi, Tokyo, Japan), followed by coating with osmium using an ion sputter machine (HPC-20; Vacuum Device, Ibraki, Japan) prior to SEM observation (S-4800; Hitachi). CellTiter-Blue^®^ Cell Viability Assay (Promega, Madison, WI, USA) was used to evaluate the cell viability. rBMMSCs cells (4 × 10^4^) were seeded on the NANOZR and FHA of the 24-well plate for 24 h. The samples were washed twice with PBS and treated with 300 μL of diluted CellTiter-Blue^®^ Reagent (50 μL CellTiter-Blue^®^ Reagent diluted in 250 μL PBS). Finally, after a 1 h incubation (37 °C; 5% CO_2_), 100 μL reagent/well was transferred to a 96-well plate and examined with the spectrometer (SpectraMax M5; Molecular Devices, San Jose, CA, USA) at 560/590 nm.

### 4.5. QRT-PCR, Alkaline Phosphatase Activity, and Calcium Deposition

Gene expression was assessed using the real-time TaqMan RT-PCR assay (Life Technologies, Carlsbad, CA, USA). Total RNA was extracted with a RNeasy Mini kit (Qiagen, Venlo, the Netherlands), and 10 µL aliquots of each RNA sample were reverse transcribed into cDNA using the Prime Script RT Reagent kit (Takara Bio, Shiga, Japan). We investigated the alkaline phosphatase (ALP) quantities and the runt-related transcription factor (Runx2) on Day 3 and Day 7, and bone morphogenetic protein 2 (BMP-2) and Bglap on Day 14 and Day 21 of the rBMMSCs. To evaluate the ALP activity, after 7 or 14 days of incubation, samples were washed with PBS, and cells that had attached to the sample surface were dissolved with 300 µL of 0.2% (*v*/*v*) Triton X-100. ALP activity was evaluated by an alkaline phosphatase luminometric enzyme-linked immunosorbent assay (ELISA) kit (Sigma-Aldrich, St. Louis, MO, USA) as per the manufacturer’s instructions. Following 21 or 28 d of incubation, calcium deposition in the extracellular matrix was measured after dissolution with 10% (*v*/*v*) formic acid. The calcium content was quantified and calculated using a Calcium E-test kit (Wako Pure Chemical Industries, Osaka, Japan) according to the manufacturer’s instructions.

### 4.6. Implantation into Rat Femurs

This protocol was approved by the Osaka Dental University Ethics Committee, Japan (approval no. 20–08004, 31 July 2020), and complied with the National Animal Care Guidelines. Sixteen male Sprague–Dawley rats weighing 180–200 g and aged eight weeks were randomly assigned to be implanted with the NANOZR or FHA-coated NANOZR. After general anesthesia and surgical sterilization, a 10 mm vertical incision was made at the knee joint of the right hind limb. The patella and joint tissues were then dissected to expose the distal femur. Subsequently, a 1.2 mm hole in the intercondylar notch was drilled using a dental bur with saline irrigation. Screws were then implanted, knee joints were reset, and incisions were sutured. Gentamicin (1 mg/kg) and buprenorphine (0.05 mg/kg) were injected for three days to prevent infection and to reduce postoperative pain, respectively.

### 4.7. Morphological Analysis

Immediately following dissection, the right femur containing the implants was kept in cold saline and scanned by a microcomputed tomography (CT) system (SkyScan1275, Bruker, Billerica, MA, USA) at 90 kV and 40 mA to investigate the effects of the newly formed bone around the implants. The bone volume fraction (BV/TV), trabecular number (Tb.N), trabecular separation (Tb.Sp), and trabecular thickness (Tb.Th) within the regions of interest (ROI; 500 μm around the implant and 2 mm below the epiphyseal line) in the CT images were determined using Morphometric software (CTAn; Bruker, Billerica, MA, USA).

After micro-CT scanning, the specimens were fixed in 70% ethanol solution for 3 d and subsequently stained with hematoxylin and eosin (H&E). Sections were analyzed by histomorphometry using a BZ-9000 digital microscope (Keyence Co., Osaka, Japan).

### 4.8. Statistical Analysis

All results are expressed as means with standard deviation and calculated using Microsoft Excel. The data were analyzed using the student’s *t*-test for comparisons between groups. The differences were considered statistically significant at *p* < 0.05. 

## 5. Conclusions

The NANOZR substrate with an FHA film proposed in this study is an excellent candidate for increasing the osseointegration of implants that are in contact with bone. Although the bare NANOZR shows excellent esthetic outcomes, good aging stability (as shown by its resistance to LTAD), and optimum mechanical properties, using PLD to deposit a coating of FHA on the NANOZR surface results in even more promising characteristics for implant fabrication compared to the unmodified NANOZR.
